# Predictors of anaemia incidence and remission among adolescents: longitudinal evidence from four low- and middle-income countries

**DOI:** 10.7189/jogh.16.04224

**Published:** 2026-06-19

**Authors:** Shuangyu Zhao, Sachin Shinde, Ourohiré Millogo, Rutuja Patil, Nega Assefa, Mary Mwanyika-Sando, Hanxiyue Zhang, Lina Nurhussien, Amani Tinkasimile, Wafaie W Fawzi, Kun Tang

**Affiliations:** 1 Department of Nutrition, University of California, Davis, Davis, USA; 2 Department of Global Health and Population, T. H. Chan School of Public Health, Harvard University, Boston, USA; 3 Nouna Health Research Center, Nouna, Burkina Faso; 4 KEM Hospital Research Centre, Pune, India; 5 College of Health and Medical Sciences, Haramaya University, Addis Ababa, Ethiopia; 6 Africa Academy for Public Health, Dar es Salaam, Tanzania; 7 Vanke School of Public Health, Tsinghua University, Beijing, China

## Abstract

**Background:**

Anaemia remains a major public health challenge among adolescents in low- and middle-income countries (LMICs). However, longitudinal evidence on its incidence and remission during this critical period is limited. Most existing studies assess anaemia cross-sectionally, providing limited insight into its progression or potential reversibility. We aimed to examine the dynamics of anaemia during adolescence, estimating incidence and remission over one year and identifying sociodemographic, behavioural, nutritional, and dietary predictors of these transitions.

**Methods:**

We used data from the Africa Research, Implementation Science, and Education Network’s multi-site prospective cohort in Nouna (Burkina Faso), Harar (Ethiopia), Dar es Salaam (Tanzania), and Pune (India). Baseline assessments were conducted between November 2021 and December 2023, with follow-up approximately 12 months later. The analytical sample included 3656 adolescents aged 10–19 years. Haemoglobin was measured using HemoCue® analysers and classified according to World Health Organization standards. We used Poisson regression models to estimate risk ratios (RR).

**Results:**

Anaemia prevalence was 34.4% at baseline and 32.6% at follow-up. Among 2399 adolescents who were non-anaemic at baseline, 21.2% developed anaemia, whereas 45.6% of 1257 adolescents with anaemia achieved remission. Late adolescence (RR = 0.81; 95% confidence interval (CI) = 0.70–0.95), school attendance (RR = 0.67; 95% CI = 0.57–0.80), and frequent consumption of fruits and vegetables (RR = 0.83; 95% CI = 0.73–0.95) were associated with a lower risk of incident anaemia, whereas stunting (RR = 1.61; 95% CI = 1.33–1.96) increased the risk. Remission from anaemia was more likely among school attendees (RR = 1.49; 95% CI = 1.27–1.76), adolescents living with guardians (RR = 1.19; 95% CI = 1.05–1.35), and those who frequently consumed fruits and vegetables (RR = 1.17; 95% CI = 1.06–1.29), but less likely among adolescents with depressive symptoms (RR = 0.75; 95% CI = 0.61–0.92) and stunting (RR = 0.75; 95% CI = 0.62–0.91).

**Conclusions:**

Anaemia among adolescents in LMICs is both prevalent and dynamic. Diet quality, school participation, household stability, and mental and physical health are associated with these trajectories. National nutrition and health strategies should explicitly target adolescents, integrating dietary interventions with educational and clinical platforms to accelerate anaemia reduction.

Anaemia is a major global public health challenge, with low- and middle-income countries (LMICs) bearing a disproportionate burden [1]. Although its overall global prevalence has declined over the past three decades, the absolute number of people living with anaemia increased from 1.50 billion in 1990 to 1.92 billion in 2021 [2]. The burden is particularly severe in sub-Saharan Africa (SSA) and South Asia, with the latest Lancet report estimating the highest anaemia prevalence in 2021 in western SSA (47.4%), South Asia (43.0%), and central SSA (35.7%) [2].

Adolescents aged 10–19 years, defined by the World Health Organization (WHO) [3], undergo rapid biological, psychosocial, and behavioural transitions that substantially increase their nutritional requirements and heighten vulnerability to both nutritional deficiencies and infections contributing to anaemia [4]. Anaemia during adolescence can have multiple adverse health consequences: it impairs physical performance, reduces endurance, delays sexual and reproductive maturation, and compromises immune function [5]. In school settings, anaemia has been associated with reduced attention, lower attendance, and poorer academic achievement [6]. Among girls, anaemia before or during pregnancy increases the risk of adverse birth outcomes, including low birth weight and prematurity, thereby perpetuating intergenerational cycles of malnutrition [6]. Moreover, chronic anaemia during adolescence may interact with other nutritional deficiencies (*e.g.* vitamin B12, folate), infections, or genetic haemoglobinopathies, compounding morbidity. Despite this clear vulnerability, adolescent health and nutrition remain underprioritised in public health research and policy, especially compared to the focus on young children and pregnant women [7].

The determinants of adolescent anaemia are complex and interrelated. Iron deficiency remains the primary cause, yet inadequate intake of other micronutrients, such as folate, vitamin B12, and vitamin A, often coexist with iron deficiency [8]. Infectious diseases, including malaria, chronic helminth infections, and other parasitic or bacterial infections, also contribute by destroying red blood cells, impairing nutrient absorption, or increasing physiological demand [9]. Genetic disorders, such as sickle cell trait or thalassemia, also play a role in some populations. Menstrual blood loss in girls further increases iron requirements. Structural and contextual factors, including poverty, food insecurity, limited dietary diversity, poor sanitation, inadequate water and hygiene infrastructure, limited access to healthcare, and health education, compound the biological risks [10]. Across settings, poor diet quality, low dietary diversity, poverty, and female sex are frequently associated with adolescent anaemia, whereas the evidence for other factors, including education, residence, or some behavioural characteristics, has been less consistent [2,4]. However, the relative importance of these determinants appears to vary across regions. In many SSA countries, adolescent anaemia has been linked more consistently to infection-related exposures, including malaria and helminthiasis, along with poverty and inadequate water and sanitation conditions [1,11]. By contrast, studies from South Asia more often emphasise dietary insufficiency, low dietary diversity, gender-related nutritional disadvantage, and the effects of early marriage and adolescent pregnancy among girls [4,12]. Adolescents in LMICs are often exposed to multiple risk factors simultaneously, yet relatively few studies have compared these patterns across regions using harmonised measures, making it difficult to determine whether inconsistent findings reflect true contextual differences or methodological variation across studies.

Despite the scale and urgency of adolescent anaemia, evidence on its natural history remains scarce. Most studies are cross-sectional, offering only static estimates that cannot distinguish new, persistent, or resolved cases [13,14]. Few data sets enable longitudinal assessment of anaemia incidence or remission, or comparison across diverse LMIC settings, due to infrequent follow-up and restricted measurement of time-varying exposures [4,14]. In addition, adolescents are underrepresented in routine surveillance, and major national surveys often omit key determinants such as infection status, genetic traits, dietary diversity, menstrual history, or repeated haemoglobin measurement [14,15]. Consequently, current policies and programmes are often based on limited empirical evidence and may overlook critical age- and gender-specific vulnerabilities [16].

We used data from a community-based longitudinal study conducted through the Africa Research, Implementation Science, and Education (ARISE) Network, a collaborative platform that brings together leading African institutions and global partners to build research capacity and generate actionable evidence for public health. We aimed to describe the baseline prevalence of anaemia and characteristics among adolescents aged 10–19 years in four LMICs; quantify the incidence of anaemia among adolescents who were non-anaemic at baseline and the remission rate among those with anaemia at baseline; and identify sociodemographic, dietary, behavioural, and health-related predictors of both incidence and remission. By examining both onset and recovery, we seek to provide a comprehensive understanding of anaemia’s natural history in adolescence. Our findings could inform policymakers in designing effective, scalable, and sustainable interventions, aligning with the ARISE Network’s mission to strengthen public health systems and improve adolescent health and development.

## METHODS

### Study design and settings

We used data from the ARISE Network Adolescent Health and Well-being Longitudinal Study, a two-round, multi-country prospective cohort designed to examine the social, behavioural, and environmental determinants of adolescent health in LMICs. We restricted our analysis *a priori* to four study sites in which haemoglobin was measured at both baseline and follow-up: Nouna, Burkina Faso; Harar, Ethiopia; Dar es Salaam, Tanzania; and Pune, India. We selected these sites from the broader ARISE longitudinal study, which collected baseline data from November 2021 to December 2023 across nine sites in seven SSA countries (Nouna, Burkina Faso; Harar, Ethiopia; Shai Osudoku/Ningo Prampram, Ghana; Ibadan, Nigeria; Mtubatuba, South Africa; Dar es Salaam and Tanga, Tanzania; and Iganga, Uganda) and two Asian countries (Funan County, China; and Pune, India). Follow-up surveys were conducted approximately one year after baseline surveys. Sites were selected based on existing ARISE partnerships and represented diverse settings, including three urban sites (Tanzania, Ethiopia, and Nigeria) and seven rural sites. The detailed site-specific information is published elsewhere [13,17].

### Participants and sampling

In the ARISE study, participants were randomly recruited from Health and Demographic Surveillance Systems at each site and tracked using a unique identification number. We included participants aged 10–19 years at baseline, those with no prior or current pregnancy, those with written parental or guardian consent and participant assent (for participants aged <18 years), those with consent (for participants aged 18–19 years), and those with a commitment to reside in the study area for the study duration.

**Figure 1:**
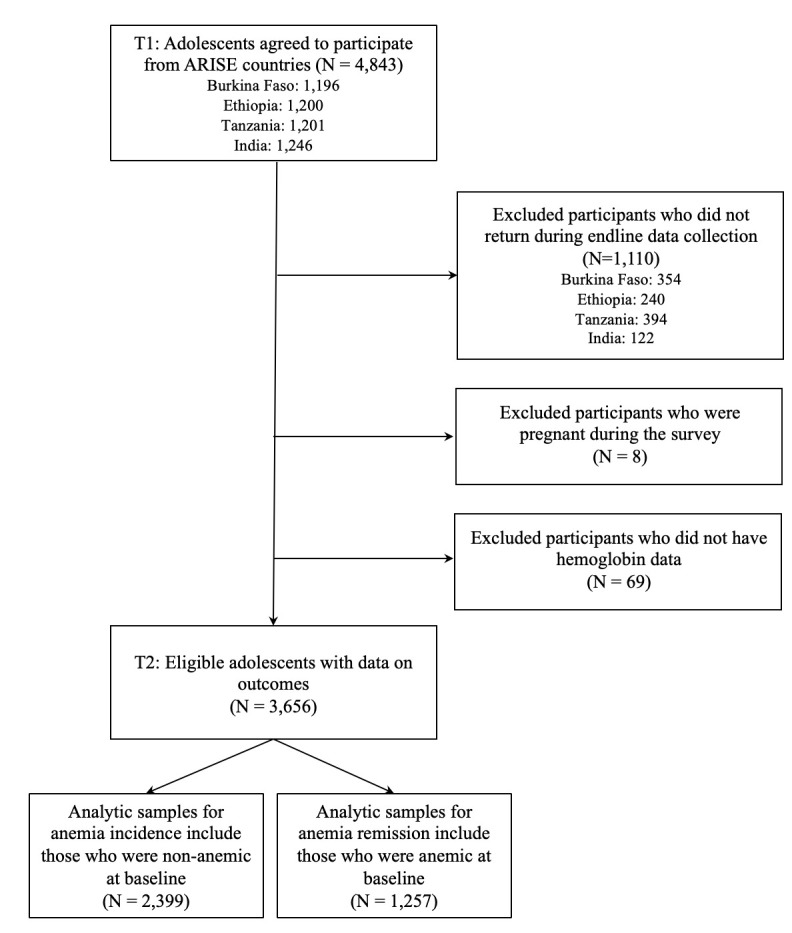
Flowchart of the sampling process. ARISE – Africa Research, Implementation Science, and Education.

A total of 4843 adolescents were enrolled and interviewed at baseline across the four sites. We excluded eight participants due to pregnancy during the survey and 69 for having missing haemoglobin data. A total of 1110 participants (22.9%) were lost to follow-up due to school dropout, migration, or non-response. The final analytical sample comprised 3656 adolescents with complete haemoglobin measurements at both time points (**Figure 1**).

### Data collection

The ARISE survey collected indicators across six domains: physical health, nutrition, sexual and reproductive health, mental health, substance use, and health services utilisation, focusing on major risk factors for preventable adolescent morbidity and mortality. Anthropometric and haemoglobin measurements followed standardised protocols. Trained interviewers conducted in-person surveys using REDCap software in English or local languages as appropriate. Further details on the survey framework and questionnaire have been previously published [13].

### Outcomes

Haemoglobin concentration was measured via finger-prick blood samples using a HemoCue® device. Anaemia was defined according to WHO sex- and age-specific thresholds: <11.5 g/dL for girls aged 10–11 years, <12 g/dL for girls aged 12–19 years, <11.5 g/dL for boys aged 10–11 years, <12 g/dL for boys aged 12–14 years, and <13 g/dL for boys aged 15–19 years [18]. All participants received an information session and a brochure on maintaining optimal haemoglobin levels. Those identified as anaemic were provided with a referral to local health facilities with documented haemoglobin and anthropometric results.

Anaemia incidence was defined as the cumulative incidence over approximately one year, which refers to the proportion of adolescents who were non-anaemic at baseline and were classified as anaemic at follow-up. Anaemia remission was defined as the proportion of adolescents who were anaemic at baseline but were no longer anaemic at follow-up.

### Exposures

Guided by the adolescent health indicator framework, the Lancet Adolescent Nutrition Series [16,19], WHO’s Global Accelerated Action for the Health of Adolescents guidance [20], prior research, and data availability, we selected 21 factors for analysis. We classified these factors into five domains: sociodemographic factors (sex, age, educational enrolment), household characteristics (living with parents/guardians, perceived socioeconomic status, source of drinking water), lifestyle factors (hand washing, mobile phone ownership, healthcare access), health conditions (history of serious injury, history of mental health symptoms, early menarche, thinness, stunting), and dietary behaviours (diet quality, consumption of animal source foods/dairy products/fruits and vegetables/roots and tubers/added fats and oils/sugar-rich foods) (Table S1 in the **Online Supplementary Document**).

### Statistical analysis

We summarised the baseline characteristics using descriptive statistics by country and the overall sample. We calculated anaemia prevalence, incidence, and remission rates as proportions with 95% confidence intervals (CI).

We analysed predictors of anaemia transitions using generalised estimating equations (GEE) with a Poisson distribution and a log link, an independence working correlation structure, and robust (sandwich/empirical) standard errors to account for within-participant correlation, allowing direct estimation of risk ratios (RR). We first estimated univariate GEE models to assess crude associations, and included variables with *P*-values of <0.05 in multivariable models. We included three additional variables (*i.e.* age groups 10–14 vs 15–19 years, sex, and site-fixed effects) regardless of univariate significance to ensure adequate control for potential confounding. We conducted separate GEE models for anaemia incidence (among adolescents non-anaemic at baseline) and remission (among adolescents with anaemia at baseline). We performed sex-stratified GEE models for anaemia incidence and remission. We assessed multicollinearity using variance inflation factors, considering values <5 acceptable.

We used STATA, version 17 (StataCorp, College Station, Texas, USA) for all analyses. We set statistical significance at a two-sided α level of 0.05.

## RESULTS

We included 3656 adolescents with complete data, corresponding to an overall follow-up rate of 75.5%, which ranged from 67.2% in Tanzania to 91.0% in India (**Figure 1**) – comparable to that reported in other voluntary adolescent cohorts [11]. Incidence of anaemia was assessed among 2399 adolescents who were non-anaemic at baseline, while remission was evaluated in 1257 adolescents with anaemia at baseline. The overall prevalence of anaemia declined from 34.4% at baseline to 32.6% at follow-up (**Table 1**), and heterogeneity was observed across the four countries, with baseline prevalence ranging from 4.8% in Ethiopia to 58.0% in Burkina Faso. The incidence of anaemia was 21.2% overall, ranging from 8.3% in Ethiopia to 44.4% in Burkina Faso, whereas 45.6% of adolescents with baseline anaemia achieved remission, with remission rates varying from 10.9% in Ethiopia to 65.6% in Burkina Faso.

**Table 1:** The prevalence, incidence, and remission of anaemia among adolescents in four ARISE countries*

	Overall (n = 3656)	Burkina Faso (n = 842)	Ethiopia (n = 960)	Tanzania (n = 734)	India (n = 1120)
**Prevalence at baseline**	1257 (34.4)	488 (58.0)	46 (4.8)	379 (51.6)	344 (30.7)
**Prevalence at endline**	1193 (32.6)	477 (56.7)	81 (8.4)	239 (32.6)	396 (35.4)
**Incident case**	509 (21.2)	157 (44.4)	76 (8.3)	71 (20.0)	205 (26.4)
**Remissive case**	573 (45.6)	168 (65.6)	41 (10.9)	211 (55.7)	153 (44.5)

ARISE – Africa Research, Implementation Science, and Education

*Values are presented as n (%).

There was an approximately equal proportion of males (49.7%) and females (50.3%) within the cohort, as well as those aged 10–14 years (52.9%) and those aged 15–19 years (47.1%) (**Table 2**). Most adolescents were enrolled in school (86.4%) and lived with parents or guardians (72.9%). Perceived household socioeconomic status was predominantly middle class (83.7%). Regarding health behaviours, most practised handwashing after toilet use (72.7%) and had accessed healthcare in the past year (81.0%). While 54.6% of participants had high diet quality, frequent consumption of animal-source foods (33.2%), dairy products (41.1%), and fruits and vegetables (43.9%) was less common.

**Table 2:** Baseline characteristics of participants by country*

	Overall (n = 3656)	Burkina Faso (n = 842)	Tanzania (n = 960)	Ethiopia (n = 734)	India (n = 1120)	*P*-value
**Sociodemographic characteristics**						
Sex						<0.001
*Male*	49.7 (48.1–51.3)	58.6 (55.2–61.8)	45.9 (42.3–49.5)	40.0 (36.9–43.1)	53.8 (50.8–56.7)	
*Female*	50.3 (48.7–51.9)	41.4 (38.2–44.8)	54.1 (50.5–57.7)	60.0 (56.9–63.1)	46.2 (43.3–49.2)	
Age in years						<0.001
*10–14*	52.9 (51.3–54.5)	61.2 (57.8–64.4)	60.4 (56.6–64.0)	34.3 (31.3–37.3)	57.8 (54.7–60.6)	
*15–19*	47.1 (45.5–48.7)	38.8 (35.6–42.2)	39.6 (36.0–43.4)	65.7 (62.7–68.7)	42.2 (39.4–45.3)	
Educational enrollment						<0.001
*Out of the education system*	13.6 (12.5–14.7)	36.7 (33.5–40.0)	11.0 (9.6–12.5)	2.9 (2.1–4.2)	7.0 (5.6–8.6)	
*Enrolled in an educational institute*	86.4 (85.3–87.5)	63.3 (59.9–66.5)	89.0 (86.5–91.0)	97.1 (95.8–98.0)	93.0 (91.3–94.4)	
**Household characteristics**						
Living with parents/guardians	72.9 (71.5–74.4)	87.5 (85.2–89.6)	73.8 (70.3–77.7)	87.7 (85.3–89.7)	49.6 (46.7–52.4)	<0.001
Perceived socio-economic status†						<0.001
*Low*	16.3 (15.2–17.6)	27.5 (24.6–30.7)	20.0 (17.2–23.2)	16.9 (14.4–19.8)	4.9 (3.9–6.3)	
*Middle and above*	83.7 (82.4–84.8)	72.5 (69.3–75.4)	80.0 (76.8–82.8)	83.1 (80.2–85.6)	95.1 (93.7–96.1)	
Source of drinking water						<0.001
*From an improved source*	23.4 (22.0–24.8)	10.9 (9.0–13.2)	4.5 (3.2–6.3)	16.0 (13.8–18.5)	51.3 (48.5–54.2)	
*From an unimproved source*	76.6 (75.2–78.0)	89.1 (86.8–91.0)	95.5 (93.7–96.8)	84.0 (81.5–86.2)	48.7 (45.8–51.5)	
**Lifestyle factors**						
Hand washing after using the toilet	72.7 (71.3–74.1)	42.4 (39.0–45.8)	47.7 (44.0–51.5)	96.4 (94.6–97.7)	91.7 (89.8–93.2)	<0.001
Not owned a mobile phone	59.0 (57.4–60.6)	81.0 (78.2–83.6)	83.7 (81.0–86.1)	39.8 (36.7–43.0)	42.8 (39.9–45.8)	<0.001
Healthcare access in the past year	81.0 (79.7–82.3)	13.5 (11.4–16.0)	48.1 (44.8–51.5)	19.2 (16.8–21.8)	3.9 (2.8–5.5)	<0.001
**Health conditions**						
Experienced severe injury within the past year	10.4 (9.5–11.5)	17.5 (15.0–20.2)	13.2 (10.9–15.9)	2.8 (1.9–4.1)	9.8 (8.2–11.7)	<0.001
Experienced mental health symptoms within the last two weeks	6.0 (5.3–6.8)	12.6 (10.5–15.0)	7.2 (5.5–9.3)	3.0 (2.1–4.3)	2.9 (2.0–4.1)	<0.001
Early menarche (n = 1840)	0.7 (0.4–1.1)	0.3 (0.0–1.3)	0.8 (0.3–2.4)	0.9 (0.4–2.0)	0.6 (0.2–1.7)	0.743
Thinness	13.5 (12.4–14.6)	9.1 (7.4–11.3)	10.6 (8.4–13.2)	10.9 (8.9–13.1)	20.8 (18.6–23.3)	
Stunted	9.5 (8.6–10.5)	12.6 (10.5–15.1)	13.3 (11.4–15.6)	5.3 (3.6–7.7)	8.2 (6.5–10.3)	
**Diet behaviours‡**						
Diet quality						<0.001
*High quality*	54.6 (53.0–56.2)	72.2 (69.2–75.1)	41.4 (38.4–44.5)	63.9 (60.8–66.9)	41.9 (38.9–44.9)	
*Low quality*	45.4 (43.8–47.0)	27.8 (24.9–30.8)	58.6 (55.5–61.6)	36.1 (33.1–39.2)	58.1 (55.1–60.9)	
Frequent animal-source food consumption	33.2 (31.6–34.7)	20.1 (17.6–22.9)	40.3 (37.3–43.5)	42.3 (39.1–45.5)	30.4 (27.8–33.2)	<0.001
Frequent dairy product consumption	41.1 (39.5–42.7)	27.2 (24.4–30.3)	20.3 (17.9–23.0)	66.4 (63.3–69.3)	43.6 (40.6–46.5)	<0.001
Frequent fruits and vegetables consumption	43.9 (42.4–45.6)	13.7 (11.7–16.1)	70.0 (66.6–73.2)	66.0 (62.9–69.1)	30.7 (27.9–33.6)	<0.001
Frequent roots and tubers consumption	28.7 (27.3–30.2)	8.4 (6.7–10.5)	52.5 (49.1–55.9)	40.7 (37.6–43.9)	18.2 (15.9–20.7)	<0.001
Frequent added fats and oils consumption	16.7 (15.5–17.9)	21.7 (19.0–24.6)	12.9 (10.8–15.5)	7.5 (5.9–9.4)	23.2 (20.8–25.8)	<0.001
Frequent sugar-rich food consumption	33.6 (32.1–35.2)	17.1 (14.7–19.8)	58.4 (55.2–61.6)	46.4 (43.2–49.6)	18.8 (16.5–21.3)	<0.001

ARISE – Africa Research, Implementation Science, and Education

*Values are presented as % (95% CI).

†Perceived socioeconomic status was assessed using MacArthur’s Scale of Subjective Social Status.

‡Dietary variables were measured using the Global Diet Quality Score.

**Figure 2:**
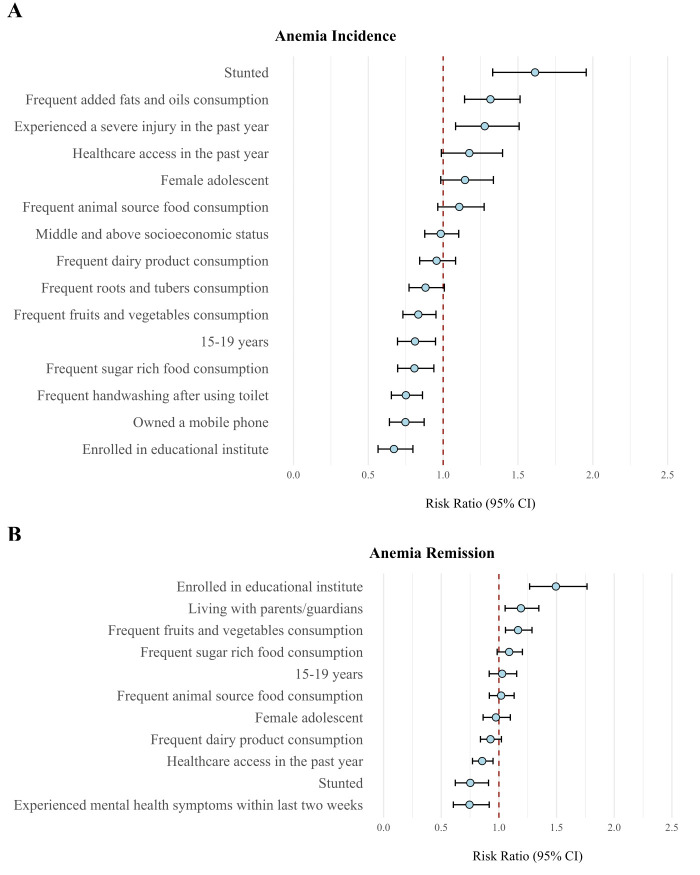
GEE results of the longitudinal association between multiple predictors and anaemia incidence and remission among adolescents. **Panel A.** Anaemia incidence. **Panel B.** Anaemia remission. CI – confidence interval.

Participants in late adolescence (RR) = 0.81; 95% CI = 0.70–0.95), those enrolled in school (RR = 0.67; 95% CI = 0.57–0.80), practicing regular handwashing (RR = 0.75; 95% CI = 0.65–0.86), owning a mobile phone (RR = 0.75; 95% CI = 0.64–0.87), and those frequently consuming fruits and vegetables (RR = 0.83; 95% CI = 0.73–0.95) or sugar-rich foods (RR = 0.81; 95% CI = 0.70–0.94) had a lower risk of developing anaemia (**Figure 2**, Panel A). Conversely, risk was higher among adolescents who experienced severe injury in the past year (RR = 1.28; 95% CI = 1.08–1.51), were stunted (RR = 1.61; 95% CI = 1.33–1.96), and frequently consumed added fats and oils (RR = 1.32; 95% CI = 1.14–1.51). Results from univariable analyses were consistent with these multivariable results (Figure S1 in the **Online Supplementary Document**).

Adolescents enrolled in school (RR = 1.49; 95% CI = 1.27–1.76), living with parents or guardians (RR = 1.19; 95% CI = 1.05–1.35), and frequently consuming fruits and vegetables (RR = 1.17; 95% CI = 1.06–1.29) were more likely to experience remission (**Figure 2**, Panel B). Conversely, remission was less likely among adolescents with mental health problems (RR = 0.75; 95% CI = 0.61–0.92), those who were stunted (RR = 0.75; 95% CI = 0.62–0.91), and those who had accessed healthcare in the previous year (RR = 0.86; 95% CI = 0.77–0.95). Results of univariable models were consistent with these multivariable findings (Figure S2 in the **Online Supplementary Document**).

**Figure 3:**
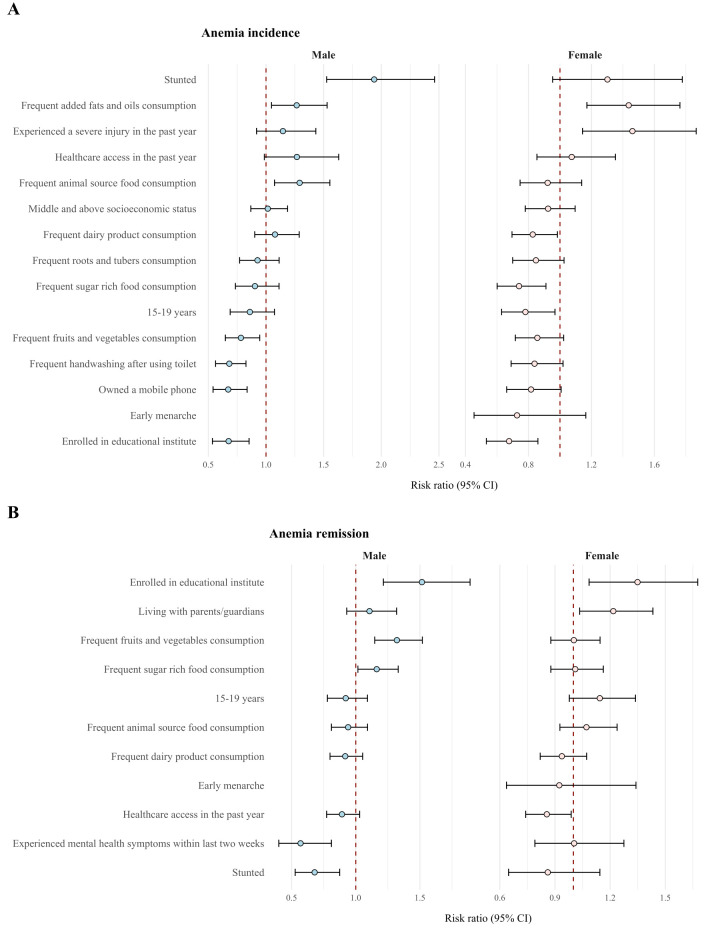
Sex-specific GEE results of the longitudinal association between multiple predictors and anaemia incidence and remission among adolescents. **Panel A.** Anaemia incidence. **Panel B.** Anaemia remission. CI – confidence interval.

Among males, the risk of developing anaemia was higher among adolescents who were stunted (RR = 1.93; 95% CI = 1.52–2.46) and those who frequently consumed added fats and oils (RR = 1.26; 95% CI = 1.07–1.52) (**Figure 3**, Panel A). Lower risk was observed among males who were enrolled in an educational institute (RR = 0.67; 95% CI = 0.54–0.82), owned a mobile phone (RR = 0.67; 95% CI = 0.55–0.81), practiced frequent handwashing after using toilet (RR = 0.68; 95% CI = 0.56–0.81), and frequently consumed fruits and vegetables (RR = 0.77; 95% CI = 0.64–0.93). Among females, incident anaemia was more likely among adolescents who frequently consumed added fats and oils (RR = 1.42; 95% CI = 1.18–1.75) and those who experienced a severe injury in the past year (RR = 1.44; 95% CI = 1.16–1.85). In contrast, lower incidence was observed among females aged 15–19 years (RR = 0.78; 95% CI = 0.63–0.97), those enrolled in an educational institute (RR = 0.69; 95% CI = 0.55–0.87), those who frequently consumed dairy products (RR = 0.83; 95% CI = 0.70–0.98), and those who frequently consumed sugar-rich foods (RR = 0.75; 95% CI = 0.60–0.91).

Among males, adolescents enrolled in an educational institute (RR = 1.52; 95% CI = 1.18–1.86), those who frequently consumed fruits and vegetables (RR = 1.30; 95% CI = 1.12–1.51), and those who frequently consumed sugar-rich foods (RR = 1.15; 95% CI = 1.02–1.29) were more likely to experience remission (**Figure 3**, Panel B). Conversely, remission was less likely among males who experienced mental health symptoms within the last two weeks (RR = 0.56; 95% CI = 0.42–0.79) and those who were stunted (RR = 0.66; 95% CI = 0.53–0.82). Among females, remission was more likely among adolescents enrolled in an educational institute (RR = 1.30; 95% CI = 1.08–1.64) and those living with parents or guardians (RR = 1.22; 95% CI = 1.02–1.41). In contrast, females who had accessed healthcare in the previous year were less likely to experience remission (RR = 0.85; 95% CI = 0.69–0.98).

## DISCUSSION

With this longitudinal study across four LMICs, we provide evidence on the dynamic nature of anaemia during adolescence. Among adolescents who were non-anaemic at baseline, the one-year incidence of anaemia was 21.2%, while among those who were anaemic at baseline, 45.6% experienced remission. These transitions highlight that anaemia in adolescence is not a fixed condition but one shaped by modifiable exposures, with important implications for targeted prevention and treatment strategies. Key determinants of both anaemia incidence and remission included dietary patterns, educational enrolment, growth indicators, and broader socioeconomic circumstances, with important implications for intervention design and public health policy.

Compared to the longitudinal study in India by Rai *et al.* [21], we observed similar prevalence, incidence, and remission rates of anaemia among adolescents, reinforcing the dynamic nature of anaemia during this life stage. Beyond these shared patterns, we contributed new evidence that frequent consumption of fruits and vegetables protects against anaemia incidence and promotes remission, while stunting both increases the risk of incidence and inhibits recovery. By extending these insights across multiple LMICs, our results showed that these determinants also underscore the importance of integrated strategies that combine dietary quality and mental health support interventions to accelerate anaemia reduction.

Sex-stratified analyses suggested that the determinants of anaemia dynamics may differ between boys and girls. Family support was found to have a stronger impact on girls’ anaemia compared to boys, which is consistent with a recent systematic review that family engagement, particularly support, can improve adherence to iron supplementation, promoting consumption of iron-rich foods among adolescent girls [22]. Girls are also more vulnerable to injury-led anaemia, as they enter adolescence with higher iron requirements due to menstruation, and a moderate blood loss during an injury pushes them immediately below the clinical diagnostic threshold for anaemia [5]. Digital exposure was associated with lower anaemia risk only among boys, which is consistent with our cross-sectional ARISE paper [23]. A potential explanation for this association could be that boys are more likely to use digital tools for education, while girls prefer to use them for social communication [24,25].

Diet quality emerged as a strong determinant of anaemia dynamics. Adolescents who frequently consumed fruits and vegetables had a lower risk of developing anaemia and a higher likelihood of remission. These findings are consistent with evidence linking dietary diversity and micronutrient adequacy to haemoglobin concentration [26,27]. Fruits and vegetables provide vitamin C, folate, and other micronutrients essential for erythropoiesis [28]. Similarly, a three-month intervention providing green leafy vegetables to school-aged children in Ghana also showed significant increases in haemoglobin levels and reductions in anaemia prevalence [29]. The consistency of this association with both incidence and remission reinforces the biological plausibility and public health importance of promoting diverse, plant-rich diets.

The inverse association between frequent consumption of sugar-rich foods and anaemia incidence, although unexpected, likely reflects socioeconomic rather than biological factors. Adolescents with greater access to sweets and processed foods may reside in households with higher food security and purchasing power, facilitating access to nutrient-rich foods. There is some evidence that simple sugars (*e.g.* fructose) can modestly enhance non-haem iron absorption when consumed with iron-containing meals [30,31]. Alternatively, higher sugar consumption may signal urban residence or market integration, where dietary diversity tends to be greater. These findings should not be interpreted as advocating increased sugar intake, given its adverse metabolic outcomes [32], but rather as a reminder to consider dietary patterns within broader socioeconomic and environmental contexts.

Frequent consumption of added fats and oils was associated with higher anaemia incidence. This may reflect energy-dense diets but micronutrient-poor, often displacing legumes, vegetables, or animal-source foods. Excess intake of low-quality fats has been associated with inflammation and altered iron metabolism in some populations [33,34]. Together, these findings highlight the complexity of dietary influences on anaemia risk and the importance of promoting balanced, nutrient-dense diets among adolescents. The strong protective effect of fruit and vegetable consumption supports expanding adolescent-focused dietary interventions in LMICs.

School enrolment was consistently protective, associated with lower anaemia incidence and greater remission. Schools serve as vital platforms for health and nutrition promotion through formal and informal education, feeding programmes, deworming, micronutrient supplementation, and access to basic health services [34–36]. The link between school attendance and anaemia remission suggests that school-based programmes may facilitate recovery through multiple channels, including improved nutrition, hygiene, and healthcare utilisation. A recent cluster randomised trial in Tanzania demonstrated that integrated school-based interventions combining meals, nutrition education, and school gardens improved adolescent diet quality [37]. These findings highlight the broader value of school retention in anaemia prevention, particularly for girls.

Stunting was associated with both increased anaemia risk and lower remission, most plausibly reflecting the cumulative effects of chronic undernutrition that impair both linear growth and haematopoiesis rather than a direct causal effect of stunting on anaemia status [10,38]. Stunted adolescents may have experienced prolonged nutrient deficiencies and recurrent infections that impair haematopoiesis. This emphasises the need for life-course approaches that address nutritional deficits from early childhood and sustain dietary adequacy during adolescence [39].

Depressive symptoms were linked to a lower likelihood of anaemia remission, highlighting the interplay of mental and physical health [40]. Depression can reduce dietary intake, adherence to treatment, and engagement with health services [41], while chronic stress may alter inflammatory pathways and iron metabolism [42]. Integrating mental health support within nutrition interventions could enhance anaemia recovery and overall well-being.

Adolescents who reported accessing healthcare in the past year were less likely to achieve remission, likely reflecting reverse causality, as those with more severe or persistent anaemia, or other underlying health conditions that influence anaemia status, such as chronic infections or inflammatory disorders, seek care more often. This may also indicate limitations in the quality of adolescent health services, which in many LMICs remain focused on maternal and child health, with limited diagnostic and treatment capacity for adolescents [43]. Therefore, strengthening routine primary care to include accurate anaemia screening, appropriate supplementation, management of infections, and follow-up care is essential [20].

Living with parents or guardians was associated with greater anaemia remission, underscoring the protective role of household stability. Family support may improve access to nutritious foods, health services, and caregiving, whereas adolescents living apart from parents may face economic insecurity and poorer dietary adequacy, highlighting the need for targeted support to vulnerable groups [44].

Our findings emphasise adolescence as a pivotal window for anaemia prevention and treatment. While early childhood remains a critical period, adolescence represents a second opportunity to improve nutritional status and long-term health [14,45]. Effective strategies should combine dietary diversification, supplementation, and fortification with actions that promote school retention and strengthen adolescent health services [16]. Schools offer scalable delivery platforms, while primary healthcare systems must be equipped to diagnose and manage anaemia among adolescents. Expanding national surveillance to include adolescent nutrition indicators will improve policy targeting and accountability [46].

This study has several strengths. We used a multi-country, prospective design, including a large and diverse sample and a harmonised data collection across settings. Measuring both anaemia incidence and remission provided a dynamic perspective on transitions that cross-sectional studies cannot capture. The use of standardised instruments for diet, health, and socioeconomic characteristics enhances comparability and external validity.

However, several limitations should be acknowledged. Haemoglobin was the only biomarker assessed, limiting the ability to distinguish iron-deficiency anaemia from other causes such as infection, inflammation, or genetic haemoglobinopathies. Dietary data were self-reported, introducing potential recall and social desirability bias. The one-year follow-up period may not capture longer-term or seasonal variations, and modest loss to follow-up could bias estimates if related to anaemia risk. To assess the potential for attrition bias, we compared key sociodemographic characteristics, including sex, age, and socioeconomic status, between participants retained at follow-up and those enrolled at baseline and observed no statistically significant differences. In addition, the cross-sectional measurement of stunting at baseline precludes any inference about the temporal sequence of these two conditions, and future studies with more granular longitudinal anthropometric and biomarker data will be needed to disentangle their relationship.

## CONCLUSIONS

Anaemia among adolescents in LMICs is widespread yet reversible. In this longitudinal study across four LMICs, nearly half of affected adolescents recovered within a year, demonstrating that improvement is achievable under supportive conditions. Key determinants of incidence and remission included dietary patterns, educational enrolment, growth indicators, and broader socioeconomic factors. National nutrition programmes should explicitly include adolescents alongside young children and pregnant women. Integrated, multisectoral strategies linking nutrition, education, mental health, and primary care can reduce the burden of adolescent anaemia and contribute to long-term human capital development.

## Additional material


Online Supplementary Document
