# Supplementary material for: Predictors of anaemia incidence and remission among adolescents: longitudinal evidence from four low- and middle-income countries
Source: J Glob Health. 2026 Jun 19;16:04224. doi: 10.7189/jogh.16.04224 (PMC13281263; doi:10.7189/jogh.16.04224)

Supplement to: Zhao S, Shinde S, Millogo O, Patil R, Assefa N, Mwanyika-Sando M, Zhang H, Nurhussien L, Tinkasimile A, Fawzi WW, Tang K. Predictors of anaemia incidence and remission among adolescents: longitudinal evidence from four low- and middle-income countries. J Glob Health. 2026;16:04224.

Table S1. Definition of factors associated with adolescent anemia incidence and remission identified from a comprehensive review of conceptual framework and prior studies.

**Factor**	**Definition**	**Reference category**
Socio-demographic factors		
Sex of adolescent	Coded as 1 if the participant reported as male; 2 if the participant reported as female.	Male
Age of adolescent	Self-reported age. Coded as 1 if the participant reported age between 10–14 years (early adolescence); 2 if the participant reported age between 15–19 years (late adolescence).	10–14 year (early adolescence)
Educational enrollment	Coded as 0 if the participant reported not in school in past 12 months; 1 if the participant reported in school.	Enrolled in educational institute
Household characteristics		
Living with parents/guardians	In the 2 following categories: (1) currently living with parents; (2) currently living with other adults or living alone.	Currently living with parents
Perceived socio-economic status	Based on the MacArthur Scale of Subjective Social Status – Youth Version (MacArthur SSS Scale),[6] ranging from 1 rung to 10 rungs. In the 3 following categories: (1) Low subjective social status (SSS), with 1–3 rungs; (2) Middle SSS, with 4–7 rungs; (3) High SSS, with 8–10 rungs. In main analysis, this variable was further classified into two group: 1) Low SSS; 2) Middle or high SSS.	Middle or high SSS
Source of drinking water	Sources of drinking water were considered as improved sources if the water was fetched from own piped water/hand pump/covered well, other piped water/hand pump/covered well, surface water/spring/river/stream/pond/rainwater, and water from a tanker, whereas open wells and other sources of water were considered as nonimproved sources of drinking water.	Drinking water from improved sources
Lifestyle factors		

Hand washing after using toilet	Frequency of hand washing with soap and water after using the toilet or latrine, in the 2 following categories: (1) never or rarely; (2) sometimes, most of the time, or always	Sometimes, most of the time, or always
Ownership of mobile phone	In the 2 following categories: (1) owned a mobile phone; (2) others, including not owned a mobile phone but can use parents' mobile phones, or cannot access to mobile phone	Owned a mobile phone
Healthcare access in the past year	Assessed using the item: "In the past 12 months, did you wish to see a health provider or have needed to use health care services, but found yourself unable to use them for some reason such as they were not available or you did not have the money?" Participants who responded "Yes" were categorized as having experienced unmet healthcare needs.	Not accessible
Health conditions		
History of serious injury within the past year	Yes if the participant had serious injury (defined as injury that made adolescents at least one full day of usual activities, or requires treatment by a doctor or nurse) in past 12 months; no otherwise	No serious injury within 12 months
History of mental health symptoms within last two weeks	Based on Patient Health Questionnaire-2 (PHQ-2), a score ranging from 1-6, in the 2 following categories: (1) had depression symptom, with score 3-6; (2) no depression symptom, with score 1-2	No depression symptoms
Early menarche	Defined as age at menarche below 12 years, based on self-reported age at first menstruation.	Menarche at ≥12 years
Thinness	Defined using WHO BMI-for-age Z-score (BAZ) < –2 SD for adolescents aged 10–19 years. Height and weight measurements were taken twice using standard weighing scales and stadiometers.	Not thin (BAZ ≥ –2 SD)
Stunted	Defined using WHO height-for-age Z-score (HAZ) < –2 SD for adolescents aged 10–19 years. Height and weight measurements were taken twice using standard weighing scales and stadiometers.	Not stunted (HAZ ≥ –2 SD)
Diet behaviors		
Diet quality	Based on Global Diet Quality Score (GDQS), a population-based metric of diet quality. The GDQS consists of 25 food groups, including 16 healthy food groups, 7 unhealthy food groups, and 2 food groups that are considered unhealthy when consumed excessively (i.e., red meat and high-fat dairy). The questionnaire assigns weighted scores to each group based on how frequently respondents consume them over the past month. The total score ranges from 0 to 49 and were	High quality

	categorized into 3 groups: (1) Low risk, with score from 23 to 49; (2) Moderate risk, with score from 15 to 23; (3) High risk, with score below 15. In main analysis, this variable was further classified into two group: 1) High quality, including moderate or high risk; 2) High quality, including low risk.	
Consumption of animal source food	Based on GDQS, using five animal source food groups: fish and shellfish, poultry and game meat, red meat, processed meat, and eggs. Dairy products were excluded. Frequencies were summed and dichotomized into high vs low.	Low consumption
Consumption of dairy products	Based on GDQS, including both low-fat and high-fat dairy groups (converted to milk equivalents when appropriate). Frequencies were summed and dichotomized into high vs low.	Low consumption
Consumption of fruits and vegetables	Based on seven GDQS food groups: citrus fruits, deep orange fruits, other fruits, dark green leafy vegetables, cruciferous vegetables, deep orange vegetables, and other vegetables. Frequencies were summed and dichotomized.	Low consumption
Consumption of roots and tubers	Based on GDQS food groups for deep orange tubers and white roots and tubers. Frequencies were summed and dichotomized into high vs low.	Low consumption
Consumption of added fats and oils	Based on the GDQS food group for liquid oils. Frequency of consumption was dichotomized into high vs low.	Low consumption
Consumption of sugar rich foods	Based on GDQS food groups for sweets and ice cream, sugar-sweetened beverages, and juice. Frequencies were summed and dichotomized into high vs low.	Low consumption

Table S2.

	**Nonanemic at endline**	**Anemic at endline**
**Nonanemic at baseline**	1,890	509
**Anemic at baseline**	573	684

Figure S1. Results of GEE to assess the longitudinal association between unique predictor and anemia incidence among adolescents in four ARISE countries at pooled level, risk ratio (95% confidence interval).

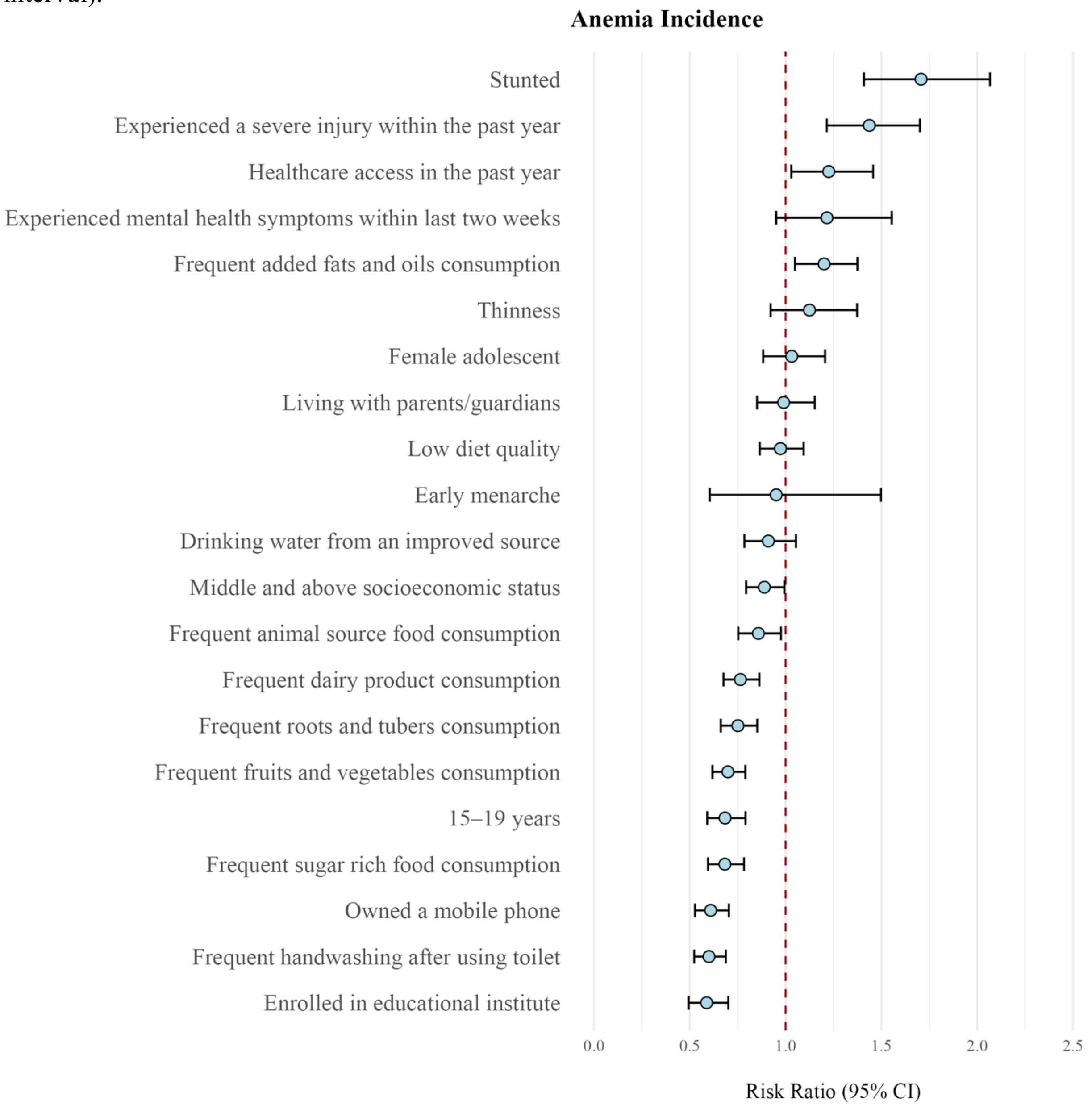

Figure S2. Results of GEE to assess the longitudinal association between unique predictor and anemia remission among adolescents in four ARISE countries at pooled level, risk ratio (95% confidence interval).

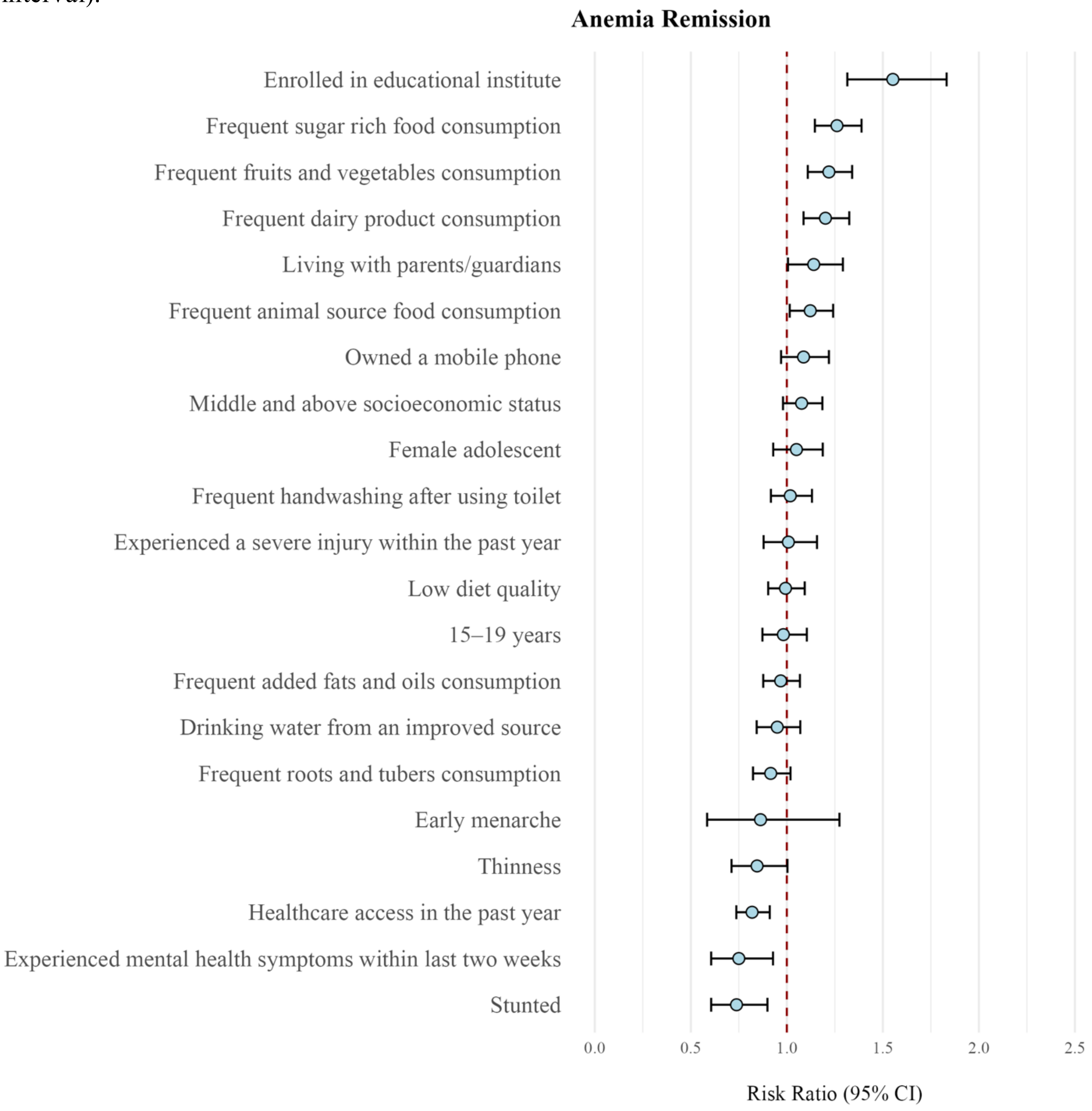

Supplement: Online Supplementary Document [file jogh-16-04224-s001.pdf]
